# A Case Report of the Ongoing Management and Comorbidities of Loin Pain Hematuria Syndrome with an Unusual Presentation

**DOI:** 10.7759/cureus.21531

**Published:** 2022-01-23

**Authors:** Casey Meier, John Grove, Adam Gromley, Rachel Bowman

**Affiliations:** 1 Research, Lincoln Memorial University DeBusk College of Osteopathic Medicine, Knoxville, USA; 2 Family Medicine, Tennova Medical Group, Knoxville, USA

**Keywords:** loin pain, lphs, chronic pain, recurrent hematuria, loin pain hematuria syndrome

## Abstract

Loin pain hematuria syndrome (LPHS) is a rare chronic pain disorder that is poorly understood. LPHS presents as unilateral or bilateral flank pain with hematuria of unknown cause. The lack of knowledge surrounding pathogenesis and effective treatment has resulted in missed diagnoses as well as narcotic addiction in some patients. In this case, we describe the presentation and management of a 30-year-old female with a history of anxiety, depression, chronic pelvic bleeding, and pain recently diagnosed with LPHS after a total hysterectomy. She presented with ongoing pelvic pain symptoms with recent tachycardia, recurrent urinary tract infections, and nephrolithiasis. Loin pain hematuria presents as a particularly rare and difficult diagnosis to manage with multiple, sometimes unpredictable, comorbidities. This case serves as an example of a unique presentation with additional uncommon symptoms.

## Introduction

Loin pain hematuria syndrome (LPHS) is a rare (0.012%) cause of recurrent hematuria. It typically presents with hematuria and severe intermittent or continuous pain located near the costovertebral angles that may radiate to the abdomen, inguinal, or thigh area [1-2]. The symptoms associated with LPHS vary in both frequency and duration. LPHS primarily affects women and can be divided into primary LPHS and secondary LPHS. Primary LPHS occurs by itself, while secondary LPHS is when symptoms are found to be associated with a glomerular disease [3]. 

The pathophysiology of LPHS is not well understood. The unknown mechanism of this disorder makes diagnosis and treatment especially difficult. There are many working theories, however, the most likely etiologies of LPHS are immunoglobulin A (IgA) nephritis, thin glomerular basement membrane disease, and intratubular microcrystal formation [4]. Other research studies suggest that LPHS may be caused by coagulopathies, hypersensitivity reactions, or renal vasospasm [4-5]. Intratubular crystal formation has been identified in roughly half of the patients diagnosed with primary LPHS; leading researchers believe the microcrystal deposition can be linked to the underlying cause of LPHS [6-7]. 

Diagnosis can be challenging due to a lack of definitive testing and unclear pathogenesis. LPHS remains a diagnosis of exclusion relying on clinical features. Other differential diagnoses may include coagulopathies, factitious disorder, nephrolithiasis, renal cysts, tumors, and glomerular disease. Kidney biopsy may reveal minor or normal pathology with retained kidney function with or without the presence of recurrent nephrolithiasis or urinary tract infections [2, 6]. If LPHS is suspected, 24-h urine collection for calcium, oxalate, citrate, uric acid, and creatinine should be collected, as roughly half of the patients with LPHS will have microcalcifications [3]. CT should be done to assess for kidney stones and ureteroscopy can be done to rule out ureteral pathologies [4]. Renal nutcracker syndrome, or compression of the left renal vein, can also be ruled out with CT [4]. Coagulation studies should be done to rule out possible coagulopathies that may be contributing to the symptoms [4]. If LPHS is suspected, the clinician should utilize a psychiatric evaluation for the possible factitious disorder [8].

The literature lacks substantial research for these patients, which makes treatment reliant on anecdotal and case reports. Here, we report an unusual presentation of LPHS in a 30-year-old female suffering from extensive obstetric medical history before a diagnosis of LPHS. Her current treatment is ongoing as there is a lack of evidence-based research available for reference. 

## Case presentation

A 30-year-old Caucasian female presented for primary care follow-up and management for chronic pelvic pain and hematuria. Over the past several years she has seen specialists in urology, nephrology, cardiology, hematology, and obstetrics/gynecology (OBGYN) to find diagnoses and treatments for a multitude of symptoms. 

Her past medical history includes dysmenorrhea starting at age nine, one miscarriage, two full-term deliveries, dilatation and curettage (D&C), impacted intrauterine device (IUD) removal with tubal ligation, dyspareunia, recurrent urinary tract infection (UTI), and gonorrhea/chlamydia infection that progressed to a pelvic inflammatory disease which ultimately resulted in an elective total hysterectomy at age 29.

In addition to her extensive obstetric history, at age 22 she passed multiple kidney stones which led to the development of hydronephrosis. Gross hematuria was noted. A UTI was also diagnosed during this time. After this episode, she continued to experience intermittent flank pain as described in her upper shoulder blades radiating down her leg. 

Following a hysterectomy, her pelvic pain and hematuria continued. She was found to have kidney stones which were treated with laser lithotripsy and stent. Both gross and microscopic hematuria in addition to pelvic pain continued following successful treatment of nephrolithiasis and recurrent UTIs. Further investigation with cystourethroscopy revealed no lesions. Kidney function remained intact with Cr 0.7. antinuclear antibodies (ANA), antineutrophil cytoplasmic antibodies (ANCA), c3, and c4 all reported normal. Renal biopsy was normal. Multiple abdominal CT with and without contrast (CT urography) ruled out renal nutcracker syndrome, obstruction, and masses. Abdominal ultrasound and magnetic resonance urography were negative for relevant pathology. A coagulation study showed no abnormalities. The patient did have recurrent UTIs that were appropriately treated with antibiotics. In the absence of UTI, confirmed with urinalysis, culture, and cytology, her symptoms of hematuria and flank pain continued. Interstitial cystitis is considered a possible cause, but the pain described by the patient is unrelated to the bladder specifically and does not change with regard to urination. The primary symptom of persistent gross hematuria with lower back pain in the absence of another identifiable cause leads us to the diagnosis of LPHS. 

In addition to her chronic pain management regimen, the patient has been treated with fluoxetine and alprazolam for depression and anxiety. Other medications include amitriptyline, cholecalciferol, cimetidine, cyanocobalamin, metoprolol succinate, oxycodone, and promethazine. 

Recently she has reported tachycardia. This is being monitored by an implanted loop recorder and treated with ivabradine. 

At this time, the 30-year-old female had gross hematuria, pelvic pain, abdominal swelling, dyspareunia, urinary frequency, depression, and anxiety. Physical exam reveals mild abdominal distension without evidence of fluid wave or firmness to palpation. Bowel sounds are auscultated in all four quadrants. The patient reports no tenderness to palpation of the abdomen. The urine sample reveals gross hematuria. 

## Discussion

Loin pain hematuria syndrome is a chronic, rare condition primarily affecting young females that causes severe flank pain with hematuria [4-5]. Due to the unknown etiology, LPHS can be difficult to diagnose and manage. Patients can present with a multitude of symptoms including a psychiatric component that may even cause misdiagnosis of somatization disorder in some cases [9]. 

Atypical presentations may cause more challenges in identifying patients with LPHS. In this case, a 30-year-old female presented with extensive obstetric history escalating to total hysterectomy before discovering the hematuria from the urinary tract and pelvic flank pain remained. Extensive anxiety and depression in combination with severe chronic pain may contribute to frequent hospital visits and escalating interventions in the management of this patient. The challenge of overseeing the wellness of LPHS patients before and after diagnosis is in part due to the lack of literature surrounding cause and treatment. 

Many patients suffering from LPHS have become addicted to narcotics [4, 7]. In an attempt to avoid this, other management strategies have been attempted to reduce pain and hematuria symptoms. Some medications such as angiotensin-converting enzyme (ACE) inhibitors and phosphodiesterase type 5 (PDE5) inhibitors may show promise in decreasing the frequency and severity of episodes of loin pain and hematuria [3, 8, 10]. The use of tadalafil in one study suggests ureter spasm may be involved in the pathogenesis of LPHS [11].

In addition to identifying and treating the underlying cause of LPHS, clinicians also are tasked with constructing a proper pain management plan. Many patients receive daily opioids in between pain exacerbations. Frequent hospitalizations are typically observed due to severe pain, but LPHS has not been related to increased mortality [1]. Invasive strategies have also been developed including surgical renal denervation, renal auto-transplantation, and radio-frequency ablation [4-5]. In some cases, these interventions have been successful in reducing pain episode occurrence [3-5, 9]. With the lack of randomized control trials, surgical intervention is generally avoided unless other less invasive strategies have been unsuccessful. 

Tachycardia has not been reported previously in relation to LPHS, however, this patient recently began having palpitations which have been treated with implanted loop recorder and ivabradine. It is unclear of the potential relation to the other symptoms in this case. 

The multitude of presenting symptoms related to LPHS has resulted in decreased quality of life, recurrent hospitalizations, specialist appointments, and potentially a delayed diagnosis resulting in an extensive obstetric intervention that has contributed to the ongoing challenge in managing this patient. The treatment of this patient is ongoing, but we hope to bring light to a unique case of this rare disorder for further knowledge and awareness of LPHS. 

## Conclusions

Loin pain hematuria syndrome can be difficult to both diagnose and manage due to the chronic pain and multitude of symptoms as well as the anxiety related to this illness. Here we report a case of a 30-year-old female presenting with an extensive obstetric history with new tachycardia in addition to persistent loin pain and hematuria. Further reporting of case presentations is needed to ensure early diagnosis as well as an understanding of the pathophysiology and treatment of the rare condition of LPHS. 
